# Protein arginine methylation in transcription and epigenetic regulation

**DOI:** 10.3389/freae.2023.1245832

**Published:** 2023-08-04

**Authors:** Hoang Quoc Hai Pham, Xiaoqun Tao, Yanzhong Yang

**Affiliations:** ^1^ Department of Cancer Genetics and Epigenetics, Beckman Research Institute, City of Hope National Cancer Center, Duarte, CA, United States; ^2^ Irell and Manella Graduate School of Biological Sciences, Beckman Research Institute, City of Hope National Cancer Center, Duarte, CA, United States

**Keywords:** post-translational modification, arginine methylation, transcription, epigenetics, histone modification

## Abstract

Arginine methylation is a prevalent post-translational modification found in all eukaryotic systems. It involves the addition of a methyl group to the guanidino nitrogen atoms of arginine residues within proteins, and this process is catalyzed by a family of enzymes called protein arginine methyltransferases (PRMTs). In mammals, there exist nine PRMTs (PRMT1–9) that catalyze three distinct types of arginine methylation: monomethylarginine, asymmetric dimethylarginine, and symmetric dimethylarginine. These modifications play critical roles in numerous fundamental cellular processes, including transcription, RNA metabolism, genome maintenance, and signaling transduction. Aberrations in protein arginine methylation have been implicated in various human diseases, such as neurodevelopmental disorders and cancer. This review offers a general overview of arginine methylation, covering its deposition, its impact on protein function, and the diverse regulatory mechanisms involved. We specifically focus on an in-depth view of the role of arginine methylation in transcription and the epigenetic regulation of gene expression. Readers are directed towards additional reviews that encompass other aspects of arginine methylation biology.

## Introduction

Among the twenty amino acids, arginine has a distinguishing feature for its positively charged guanidino group. This unique moiety allows arginine to engage in molecular interactions by forming up to six hydrogen bonds, and its chemical reactivity enables a wide array of chemical modifications (Lassak et al., 2019). These modifications, including methylation, phosphorylation, and ADP-ribosylation, in turn, play a crucial role in regulating the physiological properties of arginine-mediated molecular interactions. In eukaryotic cells, methylation is the most abundant modification found on arginine residues. It is estimated that about 2% of arginine residues are methylated in rat liver nuclei (Boffa et al., 1977), while mammalian tissues contain approximately 0.5% of arginine residues in a methylated state (Matsuoka, 1972). Consequently, arginine methylation is as abundant as other widely known modifications, such as phosphorylation and ubiquitination (Khoury et al., 2011; Zhang et al., 2021).

There are three types of methylated arginine resides, namely, ω-*N* ^*G*^-monomethylarginine (MMA), ω-*N* ^*G*^ *,N* ^*G*^-asymmetric dimethylarginine (ADMA) and ω-*N* ^*G*^ *,N'* ^*G*^-symmetric dimethylarginine (SDMA). MMA is produced in the initial reaction, followed by a sequential catalytic reaction that drives further methylation resulting in dimethylarginines (Figure 1A). In mammalian cells, the abundance of ADMA modification surpasses that of MMA and SDMA combined, with an estimated ratio of 9:1 (Maron et al., 2021; Zhang et al., 2021). However, this ratio can vary depending on the specific cell types being studied and the method being used. Mounting evidence emerged from the past two decades has clearly established arginine methylation as a key regulator of protein function in various cellular processes, including transcription, RNA metabolism, genome maintenance, and signaling transduction (Bedford and Clarke, 2009; Blanc and Richard, 2017), and dysregulation of arginine methylation has been implicated in various human diseases, such as neurological disorders (Quan et al., 2015; Hofweber et al., 2018; Qamar et al., 2018; Ryan et al., 2018; Yoshizawa et al., 2018) and cancers (Yang and Bedford, 2013; Jarrold and Davies, 2019; Hwang et al., 2021). Additionally, the rapid development of small molecule inhibitors of protein arginine methylation has greatly boosted the interest of clinical and translational research to tackle arginine methylation in diseases (Kaniskan et al., 2018). In more recent years, the study of protein arginine methylation has entered a new era. This is largely benefited from the development of new tools and technologies that have provided unprecedented opportunities for detecting, monitoring, and manipulating cellular arginine methylation levels. Specifically, the combination of pan-methylarginine antibodies raised against short methylated-peptides with the advanced proteomic techniques, such as stable isotope labeling by amino acids in cell culture (SILAC) and strong cation exchange (SCX) chromatography, as well as nuclear magnetic resonance (NMR), have enabled the identification of arginine-methylated protein substrates and quantification of arginine methylation levels in cell lines and mouse tissues to a prodigious scale and depth (Boisvert et al., 2003; Ong et al., 2004; Hung et al., 2009; Uhlmann et al., 2012; Guo et al., 2014; Hartel et al., 2019; Maron et al., 2021; Zhang et al., 2021). These new discoveries have provided us with an ever-evolving comprehensive view of this important PTM in cellular function. Particularly relevant to this review, proteins involved in transcription and epigenetic regulation have been identified as the most significantly enriched category among all the arginine-methylated substrates (Boisvert et al., 2003; Guo et al., 2014; Hartel et al., 2019). Thus, we focus our review on the role of arginine methylation in this area. Notably, aberrant protein arginine methylation underlies many human diseases, and significant progress has been made in the development of small molecule inhibitors of arginine methylation. We direct readers that are more interested in these topics to these recent reviews (Fuhrmann and Thompson, 2016; Blanc and Richard, 2017; Guccione and Richard, 2019; Lorton and Shechter, 2019; Tewary et al., 2019; Couto e Silva et al., 2020; Qin and Xu, 2021; Wu et al., 2021).

**FIGURE 1:**
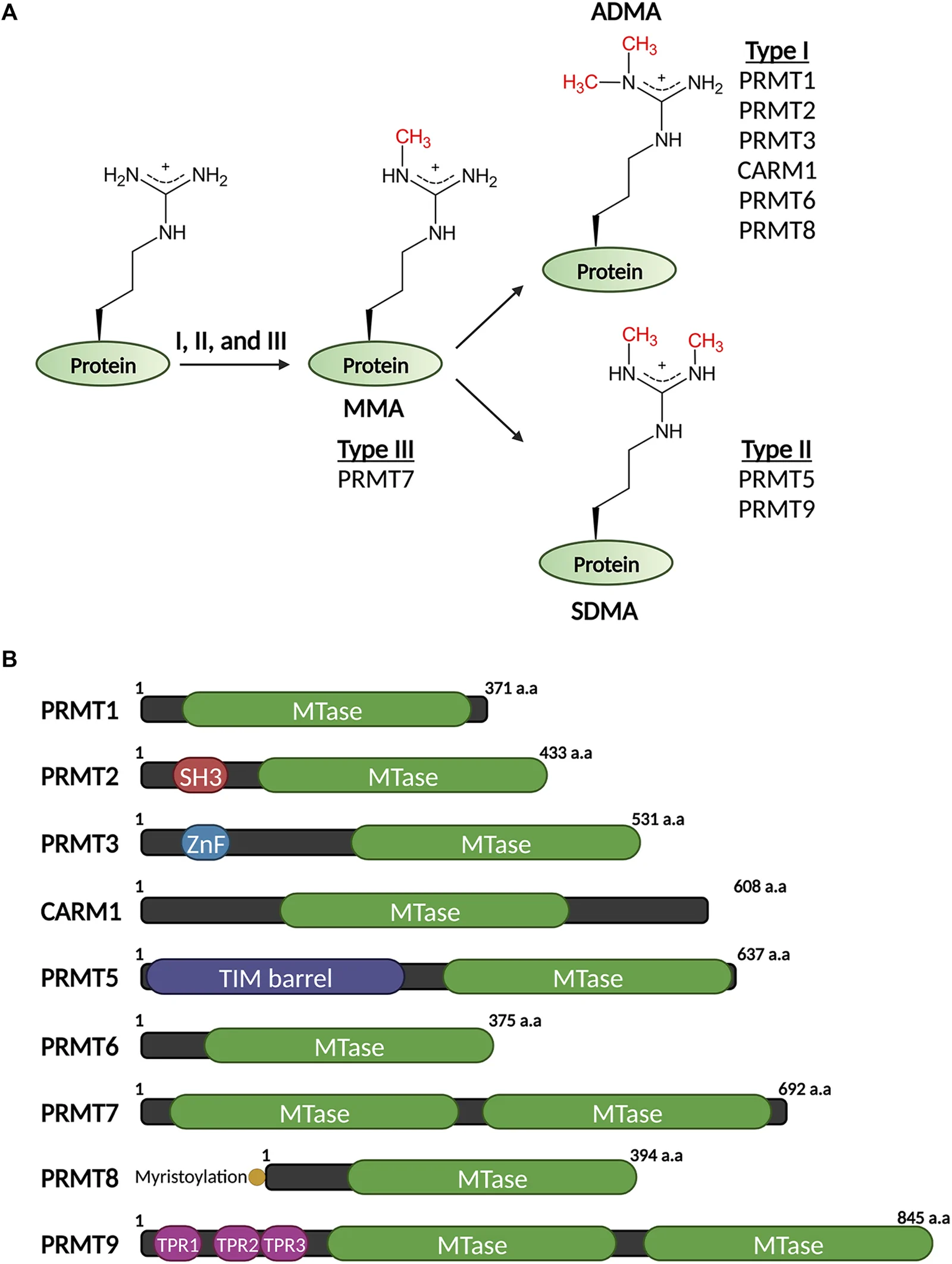
Three types of methylation on arginine residues **(A)** and domain structures of mammalian PRMTs that catalyze these modifications **(B)**.

## Writers, readers, and erasers of protein arginine methylation

### Protein arginine methyltransferases (PRMTs)

Arginine methylation is catalyzed by a family of protein arginine methyltransferases (PRMTs), also known as writers of arginine methylation. They belong to the seven-β-strand methyltransferases characterized by the twisted beta-sheet structures in their catalytic domains that contain the signature methyltransferase motifs I, post-I, II, and III. They also harbor additional “double E” (two glutamate residues) and “THW” (threonine–histidine–tryptophan) sequence motifs, which are unique to the PRMT subfamily methyltransferases (Bedford and Clarke, 2009). Like all other methyltransferases, PRMTs use S-adenosyl-l-methionine (SAM) as a methyl-donating cofactor, which is subsequently converted into S-adenosyl-l-homocysteine (SAH) after the methyl-group being transferred to the guanidino group of a peptidyl arginine residue. The X-ray crystallography of the PRMT catalytic domain revealed a doughnut-shaped homodimer structure arranged in a head-to-tail pattern (Zhang and Cheng, 2003). Human genome encodes nine PRMTs (PRMT1–9) (Figure 1B), with most of them, except PRMT7 and PRMT9 that themselves form pseudo-dimers, require the dimerization state to be functionally active (Weiss et al., 2000; Zhang and Cheng, 2003). In addition to the highly similar organization in their catalytic domains, PRMTs harbor divergent amino (N)-terminal protein–protein interaction domains and/or signaling peptides that are critical for their substrate specificities and distinct subcellular localization. For example, the N-terminus of PRMT2 contains a SRC homology 3 domain (SH3 domain), which in general mediates protein–protein interactions by recognizing the proline-rich sequence motifs on cellular proteins (Weng et al., 1995). Without the SH3 domain, truncated PRMT2 exhibits seven-fold reduction of the methyltransferase activity in comparison to the full-length enzyme (Cura et al., 2017). The N-terminus of PRMT9 contains three tetratricopeptide repeats (TPRs) in tandem that are essential for PRMT9 to interact with and methylate its protein substrate, SF3B2 (splicing factor 3B subunit 2) (Hadjikyriacou et al., 2015; Yang et al., 2015). Furthermore, PRMT8, a neuronal specific PRMT, harbors a unique myristoylation motif at its N-terminus. This short motif distinguishes PRMT8 from its closest paralog PRMT1 and targets PRMT8 to the plasma membrane, where it likely methylates a unique set of substrates (Lee J. et al., 2005; Lin et al., 2013; Penney et al., 2017).

Mammalian PRMTs can be classified into three catalytic groups based on the methylation products that they produce: type I PRMTs that produce MMA and ADMA include PRMT1, PRMT2, PRMT3, PRMT4/CARM1, PRMT6 and PRMT8; type II PRMTs that produce MMA and SDMA include PRMT5 and PRMT9, and type III PRMT PRMT7, which only produces MMA (Figure 1A). Although some PRMTs share overlapping methylation substrates and exhibit marginal specificity *in vitro*, individual PRMT can demonstrate strong substrate preference and fulfill distinct cellular functions *in vivo*. For example, PRMT1, PRMT3, PRMT6, and PRMT8 have similar substrate preferences of Arg-Gly-Gly (RGG) motif for methylation *in vitro*, but PRMT1 is the predominant PRMT that catalyzes majority (>85%) of the cellular methylation, whereas PRMT3 has a very focused methylation substrate–the ribosomal protein S2 (RPS2) (Bachand and Silver, 2004; Swiercz et al., 2005; Swiercz et al., 2007). Similarly, although PRMT5 is the major SDMA enzyme that is responsible for almost all cellular SDMA deposition, it does not methylate PRMT9’s only known substrate–the R508 of SF3B2 (Hadjikyriacou et al., 2015; Yang et al., 2015). Notably, research in the past decade using loss-of-function cell lines and genetic mouse models, as well as small molecule PRMT inhibitors has also revealed significant functional redundancies among PRMT family members, many of which compete for the same substrate/site for methylation. For example, the asymmetrical dimethylation of histone 3 arginine 17 site (H3R17me2a), an active mark associated with gene promoters and enhancers, is deposited by the coactivator associated arginine methyltransferase CARM1 (Bauer et al., 2002). However, knockout of CARM1 does not lead to significant loss of global H3R17me2a. It was recently showed that PRMT6 can also catalyze H3R17me2a, and this mark only diminishes upon CARM1 and PRMT6 double knockout (Cheng et al., 2020). Another pronounced example of the redundancy among different PRMTs is the observation that loss of or inhibiting the major ADMA methyltransferase PRMT1 leads to methylation substrate scavenging by the SDMA enzyme PRMT5 (Dhar et al., 2013; Zhang et al., 2021), further suggesting that different types of methylation could compete for the same substrates. Further supporting this principle, knockdown PRMT1 expression or inhibiting PRMT1 activity using small molecule inhibitors sensitizes cancer cells to PRMT5 inhibition or knockdown (Gao et al., 2019). Further exploration of this redundancy is likely to reveal cancer cell vulnerabilities that could be harnessed for therapeutic interventions.

### Methylarginine reader proteins

Arginine methylation could potentially alter protein structures, impact protein–DNA/RNA integrations, and generate docking sites for effector proteins (Figure 2). The Tudor domain-containing proteins are the “primary” readers of methylarginine modifications (Chen et al., 2011; Pek et al., 2012). Tudor domains are ∼60 amino acids in size and use conserved aromatic residues to build an “aromatic cage” and bind methylated-arginine through cation–π and π–π stacking interactions (Selenko et al., 2001; Sprangers et al., 2003; Friberg et al., 2009; Liu et al., 2010). In humans, there are at least thirty-six Tudor domain containing proteins that can be classified into methylarginine-binding and methyllysine-binding groups (Chen et al., 2011). Although it is not possible to predict the binding specificity based on their primary amino acid sequences, structure analyses suggest that the methylarginine binding Tudor domains form a relatively narrower aromatic cage than the methyllysine Tudor domains, thus, favoring the docking of the planar methyl-guanidinium group of the arginine (Liu et al., 2012). A “common” mechanism of action for the Tudor domain containing proteins is to function as a scaffold that links methylated arginine or lysine marks to downstream effectors with specific catalytic activities, so that the methylation signal can be interpretated or transduced in support of cellular function (Pek et al., 2012). This working model is well exemplified by the Tudor domain containing protein 3 (TDRD3), which harbors a Tudor domain at C-terminus that reads active methylarginine histone marks and an oligonucleotide/oligosaccharide binding (OB) fold at N-terminus that interacts with the DNA topoisomerase 3B (TOP3B) (Yang et al., 2010; Siaw et al., 2016). TDRD3 recruits TOP3B to chromatin regions enriched for active methylarginine histone marks to resolve the DNA negative supercoiling in the wake of transcribing RNA polymerase II (RNAPII) to facilitate transcription elongation (Yang et al., 2014; Zhang et al., 2019). Similarly, the Tudor domain of another methylarginine reader protein SMN (survival of motor neuron) can recognize SDMA mark on the C-terminal domain (CTD) of RNAPII deposited by PRMT5, and concomitantly, recruits RNA/DNA helicase Senataxin to promote transcription termination (Yanling Zhao et al., 2016).

**FIGURE 2:**
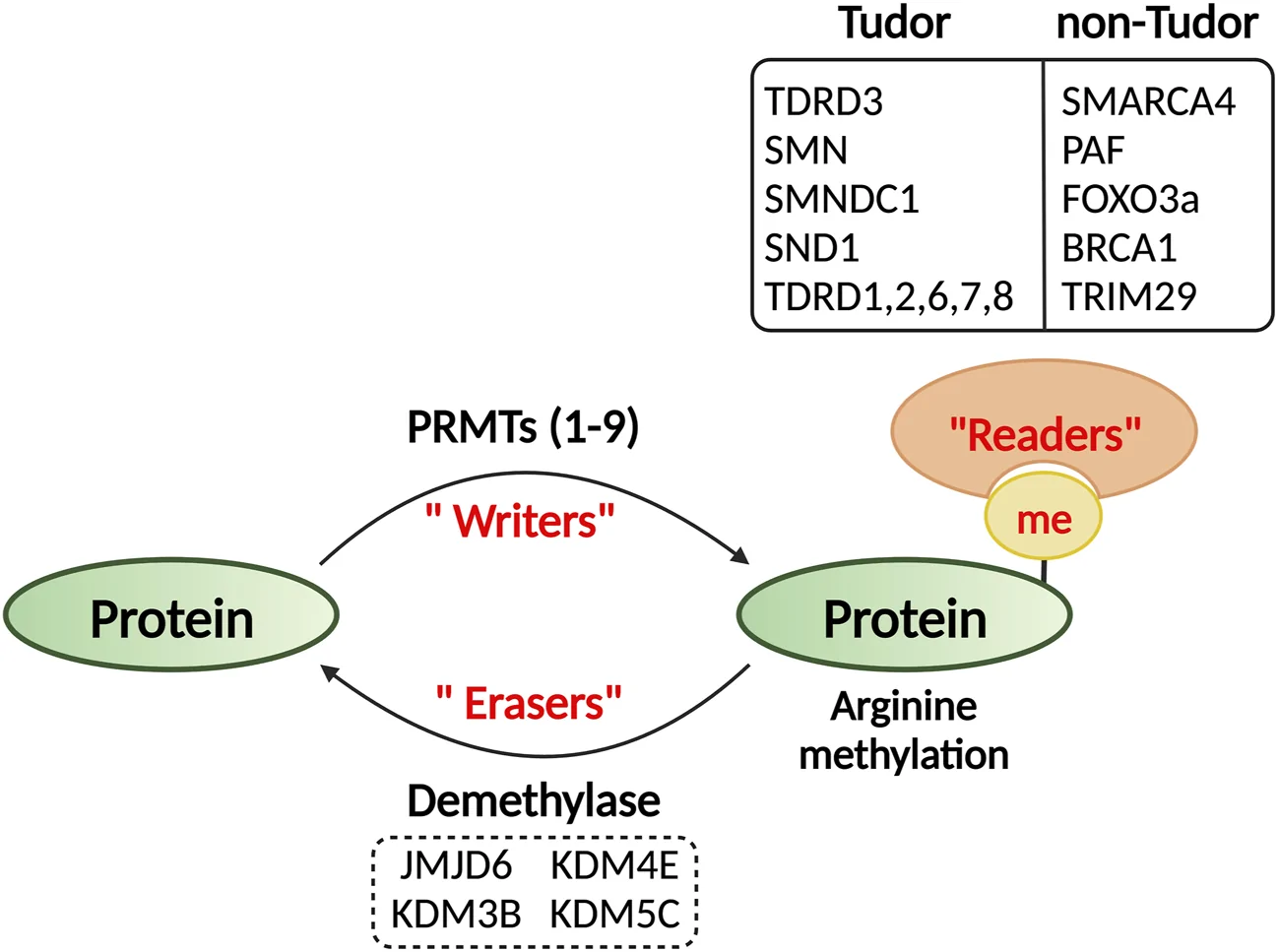
Writers, Readers, and Erasers of protein arginine methylation.

In addition to these structurally defined Tudor domain containing reader proteins, several other domains and protein folds from specific proteins also exhibited enhanced affinity towards methylated arginine residues, such as the BRCT domain of BRCA1 (Lee et al., 2011), the HELICc domain of SMARCA4 (Yao et al., 2021), the CR3 domain of FOXO3a (Yu et al., 2020), and the C-terminal domain of TRIM29 (Gao et al., 2023). Although the structural basis underlying their interactions with methylated arginine has yet been defined, the involvement of aromatic residues strongly indicates the putative aromatic cage formation for methyl-binding.

### Demethylases/erasers of arginine methylation

Once considered a stable mark, protein arginine methylation on both histone and non-histone protein substrates was recently found to be dynamic (Sarmento et al., 2004; Le Romancer et al., 2008; Litt et al., 2009), indicating the existence of an arginine demethylase or demethylases (Figure 2). However, the identification of a bona fide arginine demethylase is still under debate. The Jumonji domain containing 6 (JMJD6) was identified as the first candidate arginine demethylase for histone methylarginine marks (Chang et al., 2007). However, it was later characterized as a lysine hydroxylase that catalyzes lysyl-5-hydroxylation of the splicing factor U2 small nuclear ribonucleoprotein auxiliary factor 65-kDa subunit (U2AF65) (Webby et al., 2009). Nevertheless, recent biochemical studies using recombinant enzymes and methylated peptides suggested that the enzymes that remove methyl groups from arginine residues belong to the oxygen-sensitive dioxygenase family (Herr and Hausinger, 2018; Islam et al., 2018). For instance, biochemical analyses of a few JmjC domain-containing demethylases, including the well-defined histone lysine demethylases KDM4E and KDM5C, demonstrated that they are also capable of removing methyl groups from arginine-methylated histone peptides (Walport et al., 2016). The first evidence of arginine demethylase activity *in vivo* was reported on the H3K9me2 demethylase, JMJD1B (KDM3B), which can also catalyze demethylation of symmetrical dimethylation at histone 4 arginine 3 (H4R3me2s) (Li et al., 2018). More recently, KDM5C was reported to demethylate the autophagy activating kinase ULK1 at R170me2s deposited by PRMT5 (Li et al., 2022). It is likely that more evidence supporting arginine demethylation will emerge, whereas distinguishing the individual roles of these dual lysine and arginine demethylases remains a significant challenge.

## How arginine methylation regulates protein function?

At physiological pH, arginine is positively charged and hydrophilic. It has the potential to form up to six hydrogen bonds (Bedford and Clarke, 2009). Thus, arginine residue *per se* has great capability for mediating molecular interactions with either nucleic acids or proteins. Arginine methylation does not alter the cationic charge of the arginine residue but removes its potential hydrogen bond donors and imparts hydrophobicity of the protein substrates (Tripsianes et al., 2011), thus, could potentially alter protein structures, affect protein–protein and protein–DNA/RNA interactions.

### Protein–protein interaction

The best illustration of how arginine methylation affects protein–protein interaction is demonstrated by the interaction with Tudor domains, as well as protein sequences that have the potential to form aromatic cages (Chen et al., 2011; Wang and Bedford, 2023). This has been discussed in the previous section. Beyond the reader mediated interactions, arginine methylation can both negatively and positively regulate protein-protein interactions. For example, PRMT4/CARM1 catalyzed arginine methylation of pyruvate kinase M2 (PKM2) at R445 and 447 sites promotes the tetramerization of this key glycolytic enzyme, which greatly enhances its kinase activity (Abeywardana et al., 2018). The methylation of arginine residues neighboring proline-rich motifs in Sam68 protein prevents its association with SH3 (Src homology 3) domains, but not with WW domains (Bedford et al., 2000). Additionally, histone H3R2 is critically involved in interactions with several H3 N-terminal tail binding domains, such as PHD and WD40 (Couture et al., 2006; Taverna et al., 2007), and PRMT6 catalyzed H3R2 methylation (H3R2me2a) blocks the binding of these H3 N-terminal binders (Iberg et al., 2008).

### Protein–DNA/RNA interaction

Proteomic studies have revealed that arginine methylated proteins are highly enriched in the nucleus and RNA-binding proteins (RBPs) form the largest group of arginine-methylated substrates (Boisvert et al., 2003; Guo et al., 2014; Geoghegan et al., 2015). In principle, the positive charge of an arginine residue promotes its interaction with negatively charged nucleic acids via electrostatic interactions. Addition of a methyl group, although does not change the positive charge, could impact the interactions between protein and DNA/RNA either positively or negatively. For example, PRMT1 catalyzed arginine methylation of FMRP (Fragile X Messenger Ribonucleoprotein) at its RGG motif region reduces its RNA binding capacity (Denman, 2002; Stetler et al., 2006), whereas arginine methylation of the RNA m^6^A methyltransferase METTL14 at its C-terminal RGG motif enhances its interaction with RNA substrates (Wang et al., 2021). The molecular basic underlying this differential regulation remains unclear, but some evidence suggests that the formation of RNA secondary structures might be a consideration (Vasilyev et al., 2015; Yoshida et al., 2022).

### Arginine methylation crosstalk with other PTMs

The best characterized crosstalk between arginine methylation and other types of PTM is with phosphorylation at the consensus Akt substrate motif, RXRXXS/T (Obata et al., 2000). Demonstrated both in a nuclear transcription factor FOXO1 and a cytosolic BCL-2 antagonist of cell death (BAD), PRMT1-catalyzed methylation of arginine residues in this motif blocks Akt-mediated phosphorylation of downstream Serine (Yamagata et al., 2008; Sakamaki et al., 2011). Furthermore, the SRGG motif seems to be another hotspot of methylation–phosphorylation crosstalk (Lenard et al., 2021). Yeast Npl3p has six repeats of SRGG motif in its disordered region, which can be arginine methylated and phosphorylated (Gilbert et al., 2001; Hart-Smith et al., 2012). In this scenario, phosphorylation of Serine by the yeast kinase Sky1p inhibits the arginine methylation by the only yeast PRMT, Hmt1p (Smith et al., 2020). It is likely that this inhibitory effect is mutual, as arginine methylation of CIRBP-RGG could suppress its phosphorylation at neighboring serine by the serine-arginine protein kinase 1 (SRPK1) (Lenard et al., 2021). As these modifications are often located at the intrinsically disordered regions, it is likely that they could involve in the regulation of the biophysical property of the protein, such as phase separation. Additionally, arginine methylation could also crosstalk with other PTMs, such as acetylation (Yue et al., 2007; Yi et al., 2017) and lysine methylation (Kirmizis et al., 2007; Casadio et al., 2013).

### Arginine methylation and phase separation

Most PRMTs prefer RG/RGG motif as methylation substrates (Boisvert et al., 2003; Guo et al., 2014; Geoghegan et al., 2015). Importantly, this motif is often found at the low complexity domain or disordered region of a protein, which are critically involved in liquid-liquid phase separation (LLPS) (Rajyaguru and Parker, 2012; Thandapani et al., 2013; Chong et al., 2018; Chowdhury and Jin, 2023), a fundamental process involved in the organization of many cellular biomolecular condensates, such as nuclear speckles and cytoplasmic stress granules (Banani et al., 2017; Lyon et al., 2021). Thus, it is not surprising that arginine methylation has emerged as an important regulatory mechanism for LLPS (Wang et al., 2022). The first evidence demonstrating this type of regulation comes from the study of a protein called fused in sarcoma (FUS), a multifunctional RNA-binding protein involved in transcription, splicing, transport, and translation (Hofweber et al., 2018; Qamar et al., 2018). FUS undergoes physiologically reversible phase separation; however, pathogenic missense mutations in FUS disrupt this balance and could trigger disease, such as familial amyotrophic lateral sclerosis (fALS) and frontotemporal lobar degeneration (FTLD) (Hofweber et al., 2018; Qamar et al., 2018). As FUS phase separation is driven by Cation-π interaction between arginines and the tyrosines, arginine methylation was shown to suppress phase separation. Hypomethylation, which often occurs in FUS-associated FTLD, induces FUS condensation into stable hydrogels that disrupt RNP function in neurons. This negative regulation of LLPS by arginine methylation is also exemplified by a few stress granule proteins, including G3BP1, hnRNPA1, and KHDRBS1 (Gill et al., 2021; Wang et al., 2022). Interestingly, many of the Tudor domain containing proteins undergo LLPS in a dimethylarginine dependent manner (Goulet et al., 2008; Linder et al., 2008; Narayanan et al., 2017; Huang et al., 2018; Courchaine et al., 2021; Simcikova et al., 2023), suggesting that Tudor*–*methylarginine interaction could promote phase separation or modulate the dynamics of the condensates. Thus, while methylation itself may have a negative impact on the biophysical characteristics of arginine regarding phase separation *in vitro*, it can still exert a positive effect on LLPS by enhancing protein*–*protein interactions, and possibly also protein*–*RNA interactions, within a more complex *in vivo* environment. We suggest that the biological role of arginine methylation in cellular biomolecular condensates should be determined in a case-by-case basis.

## Arginine methylation in transcription

### Arginine methylation of transcription factors

Transcription factors (TFs) bind sequence-specific DNA elements to control gene expression in various cellular processes, including development, differentiation, and response to environment cues (Spitz and Furlong, 2012; Lambert et al., 2018). Arginine methylation of TFs can have significant effects on their activity, DNA binding affinity, and interaction with other co-regulators. For example, tumor suppressor p53 is arginine methylated at R333, R335, and R337 by PRMT5 (Jansson et al., 2008). Although these sites reside within p53 oligomerization domain, methylation seems to have more specific impact on p53’s function in cell cycle regulation rather than having a global “on” or “off” effect. In lymphomagenesis, this mechanism is harnessed by cancer cells to selectively suppresses expression of crucial proapoptotic and antiproliferative target genes, thereby sustaining tumor cell self-renewal and proliferation (Li et al., 2015). Another well-characterized arginine methylated TF is E2F Transcription Factor 1 (E2F1), which is subjected to ADMA and SDMA modifications by PRMT1 and PRMT5, respectively (Cho et al., 2012; Choi et al., 2019). The methylation sites, R109 by PRMT1 and R111/113 by PRMT5 are located in the RGRGR motif but were not present in other E2F family proteins. Interestingly, R109me2a and R111/113me2s impose opposing effects on E2F1 function in cell fate decision. R109me2a is induced upon DNA damage and associated with transcription activation of apoptotic gene expression, whereas R111/113me2s is associated with the activation of growth promoting genes (Cho et al., 2012; Choi et al., 2019). A more comprehensive list of TFs regulated by arginine methylation is summarized in Table 1.

**TABLE 1:** Arginine methylation of Transcription factors (TFs) and regulators (Regs).

TFs/Regs	Site	Types	PRMTs	Function	Refs
AR	R761	SDMA	PRMT5	Attenuates AR recruitment and represses AR-mediated transcription in prostate cancer	Mounir et al. (2016)
BAF155	R1064	ADMA	CARM1	Enhances transcriptional activity associated with SWI/SNF complex	Wang et al. (2014), Kim et al. (2021)
BCL6	R305	SDMA	PRMT5	Mediates full transcriptional repression by BCL6 in lymphoma cells	Lu et al. (2018)
BRD4	R179,181,183	ADMA	PRMT2 and CARM1	Promotes the chromatin recruitment of BRD4 to regulate selective transcription	Liu et al. (2022a)
CBP	R742	ADMA	CARM1	Promotes GRIP-1 dependent transcriptional activation	Chevillard-Briet et al. (2002)
	R580	ADMA	CARM1	Prevents KIX domain from interacting with KID of CREB to inhibit CREB activation	Xu et al. (2001)
CRCT2	R51,99,120,123	ADMA	PRMT6	Enhances CRCT2-CREB interaction to promote expression of genes encoding gluconeogenic enzymes	Han et al. (2014)
E2F-1	R109	ADMA	PRMT1	Regulates cell cycle progression; ADMA promotes apoptosis while SDMA favors proliferation; negative crosstalk between SDMA and ADMA on E2F-1	Cho et al. (2012), Zheng et al. (2013)
	R111, 113	SDMA	PRMT5		
ER	R260	ADMA	PRMT1	Mediates interaction between p85 subunit of PI3K and Src to mediate downstream hormone response	Le Romancer et al. (2008)
EZH2	R342	SDMA	PRMT5	Inhibits EZH2 phosphorylation and prevents its ubiquitination and degradation; or enhance its interaction with SUZ12 and facilitates H3K27me3 deposition	Li et al. (2020a), Li et al. (2020b), Li et al. (2021)
FOXO1	R248, 250	ADMA	PRMT1	Antagonizes phosphorylation by AKT to enhance oxidative-stress apoptosis	Yamagata et al. (2008)
FOXO3	R188, 249	ADMA	PRMT6	Mediates FOXO3 activation, contributing to muscle atrophy in skeletal muscle	Choi et al. (2019)
GATA3	R261	N/A	N/A	Mediates transcriptional activation of Il5 in Th2 cells	Hosokawa et al. (2015)
GATA4	R229,265,317	SDMA	PRMT5	Inhibits its acetylation to suppress hypertrophic responses in cardiomyocytes	Chen et al. (2014)
GLI1	R597	ADMA	PRMT1	Upregulates GLI1 transcriptional activity	Wang et al. (2016b)
GLI1	515, 915, 940	SDMA	PRMT5	Stabilizes GLI1 protein level	Abe et al. (2019)
HOXA9	R140	SDMA	PRMT5	Associates with proinflammatory roles by promoting HOXA9–E-selectin promoter transactivation	Bandyopadhyay et al. (2012)
IFI16	N/A	SDMA	PRMT5	Attenuates cytosolic DNA-induced IFN and chemokine expression in melanoma cells	Kim et al. (2020a)
LSD1	R838	ADMA	CARM1	Promotes the binding of LSD1 with deubiquitinase USP7 to stabilize the LSD1 protein	Liu et al. (2020b)
MBD2	RG motif	ADMA	PRMT1	Reduces its affinity for methyl-CG DNA	Le Guezennec et al. (2006), Tan and Nakielny (2006)
		SDMA	PRMT5		
MED12	R1862, 1899, 1912	ADMA	CARM1	Promotes MED12 transcriptional coactivator activity; or suppresses p21 transcription	Wang et al. (2015), Cheng et al. (2018)
MyoD	R121	ADMA	PRMT1	Enhances its DNA binding activity and transactivation for MyoD-mediated myogenin	Liu et al. (2019)
NFIB	R388	ADMA	CARM1	Maintains open chromatin states in tumors	Gao et al. (2023)
NFκB (p65)	R30	SDMA	PRMT5	Increases affinity and stabilizes p65 interaction for DNA to promote transcriptional activation	Wei et al. (2013)
	R30	ADMA	PRMT1	Inhibits the binding of RelA to DNA and represses NF-κB target genes in response to TNFα	Reintjes et al. (2016)
p300	R754	ADMA	CARM1	Mediates its interaction with BRCA1, functions together with p53 to induce gene expression of cell cycle	Lee et al. (2011)
	R2142	ADMA	CARM1	Inhibits p300 interaction with GRIP1	Lee et al. (2005b)
p53	R333, 335, 337	SDMA	PRMT5	Alters transcription properties of p53 in cell cycle regulation	Jansson et al. (2008)
PAX3	R271	SDMA	PRMT5	Regulates its localization to mitotic chromosomes	Wu et al. (2015)
PAX7	R10,13,22,37	ADMA	CARM1	Enhance gene expression of Myf5 via recruitment of MLL1/2	Kawabe et al. (2012)
p/CIP	R1178, 1184, 1195	ADMA	CARM1	Enhances p/CIP degradation and impairs its interaction with CBP, resulted in transcriptional repression	Naeem et al. (2007)
PGC1α	R665,667,669	ADMA	PRMT1	Enhances its coactivation function on genes associated with mitochondrial biogenesis	Teyssier et al. (2005)
	R548, 753	MMA	PRMT7	Occurs below physiological temp	Lubrino et al. (2022)
Pontin	R333,339	ADMA	CARM1	Together with the recruitment of Tip60 to activate autophagy genes that are regulated by FOXO3a	Yu et al. (2020)
RACO1	R98, 109	ADMA	PRMT1	Mediates it ubiquitination and enables its interaction with c-Jun to promote transcriptional activity by c-Jun/AP1	Davies et al. (2013)
RB	R787	ADMA	CARM1	Promotes its phosphorylation to enhance E2F-1 dependent transcription	Kim et al. (2015)
RFX5	AT-hook	ADMA	PRMT6	Downregulates selective MHC-II isotypes	Stavride et al. (2013)
RIP140	R240, 650, 948	ADMA	PRMT1	Suppresses its corepressor activity and promotes its nuclear export	Mostaqul Huq et al. (2006)
RNAPII	R1810	ADMA	CARM1	Promotes transcriptional expression of selective RNAs	Sims et al. (2011)
	R1810	SDMA	PRMT5	Mediates efficient transcriptional termination	Yanling Zhao et al. (2016)
RUNX1	R206, 210	ADMA	PRMT1	Enhances RUNX1 transcriptional activity in T cells by inhibiting its co-repressor binding	Mizutani et al. (2015)
SOX2	R113	ADMA	CARM1	Mediates SOX2 transactivation by increasing its self-association	Zhao et al. (2011)
SOX9	R177,178,179	ADMA	CARM1	Disrupts interaction of Sox9 and beta-catenin to regulate Cyclin D1 expression	Ito et al. (2009)
SPT5	R698, 681, 696	SDMA	PRMT5	Reduces SPT5-RNAPII interaction	Kwak et al. (2003)
		ADMA	PRMT1		
SRC3	R1171	ADMA	CARM1	Represses its coactivator activity that is regulated by estrogen signaling	Feng et al. (2006)
SREBP1a	R321	SDMA	PRMT5	Stabilizes SREBP1a and promotes transcription activation and lipid biosynthesis	Liu et al. (2016)
STAT3	R688	ADMA	PRMT1	Promotes its transactivation to mediate osteosarcoma malignancy	Yang et al. (2022)
	R31	ADMA	PRMT2	Regulates leptin signaling and energy homeostasis	Iwasaki et al. (2010)
TWIST1	R34	ADMA	PRMT1	Mediates active repression of E-cadherin	Avasarala et al. (2015)

### Arginine methylation of RNAPII

The C-terminal domain (CTD) of mammalian RNAPII contains 52 heptapeptide repeats (YSPTSPS) (Hsin and Manley, 2012) and the R1810 located in repeat 31 is methylated by PRMT4/CARM1 and PRMT5, generating ADMA and SDMA marks, respectively (Mizutani et al., 2015; Yanling Zhao et al., 2016). Importantly, these two types of methylation recruit different reader proteins that transduce the methylation signal to distinct downstream effects. Reading of R1810me2a by TDRD3 is proposed to recruit the DNA topoisomerase 3B (TOP3B) to resolve negative supercoiling DNA generated in the wake of transcribing RNAPII (Mizutani et al., 2015), whereas reading of R1810me2s by SMN enables the association of Senataxin helicase with RNAPII for R-loop resolution and transcription termination (Yanling Zhao et al., 2016). Interestingly, R1810 could also be deiminated to citrulline by peptidyl arginine deiminase 2 (PADI2), which is important for RNAPII pause release and efficient transcription (Sharma et al., 2019). Thus, proper spatial and temporal control of RNAPII R1810 modification by methylation and citrullination could be essential for RNAPII function.

### Arginine methylation of transcription elongation factors

The initial observation of direct involvement of arginine methylation in transcription elongation is from yeast Hmt1p (the only PRMT in yeast) mutants, which exhibited reduced elongation rate and increased transcription termination at cryptic terminators (Wong et al., 2010). Mechanistically, arginine methylation of Npl3p at its RGG motif is essential for the recruitment of elongation factors, such as Tho2p, for antitermination (Wong et al., 2010). In mammals, transcription elongation factor SPT5 is methylated by PRMT1 and PRMT5, and methylation regulates its interaction with RNAPII (Kwak et al., 2003). In this case, it seems that ADMA and SDMA exhibit similar effects in suppressing SPT5–RNAPII interaction and transcription elongation. Interestingly, methylation specifically affect SPT5 interaction with serine-2 phosphorylated, but not with serine 5 phosphorylated RNAPII, which mainly enriched at gene promoters (Kwak et al., 2003). On the other hand, the activity of transcription elongation can, in turn, impact arginine methylation levels of several elongation regulators, including SPT5. Upon transcription arrest by Actinomycin D, the levels of SPT5 monomethylation decreases (Sylvestersen et al., 2014); however, the functional significance of this regulation remains unknown.

### Arginine methylation of mediators and transcription co-regulators

Mediator is a large, conformationally flexible protein complex with a variable subunit composition (Allen and Taatjes, 2015). It functions by communicating regulatory signals from DNA-binding TFs directly to RNAPII. The mediator subunit 12 (MED12) is a major substrate of PRMT4/CARM1 in transcription regulation. Multiple arginine residues of MED12 are methylated, including R1862, R1912, and R1899 (Wang et al., 2015; Cheng et al., 2018). Although it is unclear how methylation affects mediator function, the methylarginine mark did promote its interaction with reader protein TDRD3, which functions in resolving co-translational R-loops to facilitate transcription (Yang et al., 2010; Yang et al., 2014). It is worth noting that PRMT5 was also identified to associate with mediator complex using a Flag-tag affinity purification of CDK9 and CDK19 interaction proteins (Tsutsui et al., 2013). However, PRMT5 is a known common contaminant of Flag-tag based affinity purification (Nishioka and Reinberg, 2003; Mellacheruvu et al., 2013), further validation is necessary to confirm this observation. Additionally, the histone acetyltransferases CREB binding protein (CBP) and the related p300 protein, which are key transcriptional co-activators, are methylated by PRMT4/CARM1 (Chen et al., 1999; Xu et al., 2001; Chevillard-Briet et al., 2002; Lee Y. H. et al., 2005). The methylation sites are located both at the N-terminal KIX domain (Chen et al., 1999; Xu et al., 2001; Chevillard-Briet et al., 2002) and C-terminal GRIP1 binding domain (GBD) (Lee Y. H. et al., 2005). The N-terminal methylation is important for hormone-dependent transcriptional activation, whereas the methylation of GBD inhibits the interaction of GRIP1 with p300. In Testis, GBD methylation of p300 inhibits its interaction with the haploid spermatid specific coactivator, activator of CREMτ in the testis (ACT), to suppress CREMτ target gene expression (Bao et al., 2018). These studies demonstrated that methylation at different sites of a protein could have opposing effects on co-regulator function.

## Arginine methylation in epigenetic regulation

### Arginine methylation of Histones

Histones are especially enriched for arginine residues and are subjected to extensive arginine methylation (Wysocka et al., 2006; Di Lorenzo and Bedford, 2011; Huang et al., 2015; Fuhrmann and Thompson, 2016). Most of the methylation sites are located in histone tails due to their accessibility, but methylarginine can also be found in the globular domain (Table 2). Both the location and type of methylation are critical for determining how arginine methylation might impact chromatin biology, including transcription and genome stability. For example, R3 site of Histone H4 can be modified by ADMA (H4R3me2a) or SDMA (H4R3me2s) marks by PRMT1 and PRMT5, respectively (Di Lorenzo and Bedford, 2011). These two distinct marks generate opposite effects in transcription regulation: H4R3me2a is generally considered as an active histone mark that recruits at least two effector complexes, TDRD3/TOP3B and SMARCA4 SWI/SNF chromatin remodeling complex, to promote transcription activation (Wang et al., 2001; Yang et al., 2010; Yang et al., 2014; Yao et al., 2021); whereas H4R3me2s is often associated with a repressive chromatin status (Xu et al., 2010), possibly by recruiting DNMT3A for *de novo* DNA methylation (Zhao et al., 2009). Although the methylation levels of individual sites vary between tissues, cell types, developmental stages, as well as physiological vs. pathological conditions, H4R3me2a seems to be the most abundant methylarginine histone mark with ∼10% stoichiometry in adult mouse brain (Tweedie-Cullen et al., 2012). As technology continues to advance in mass spectrometry, an ever-expanding repertoire of arginine methylation sites is being discovered (Huang et al., 2015), presenting opportunities for further characterization and investigation. For example, mono- and di-methylation of H4R17 and R19 were identified at considerable level (2–8%) in mouse brain (Tweedie-Cullen et al., 2012), but their biological function is still unknown. PRMT7 has been shown to methylate both sites on a peptide substrate (Feng et al., 2013), and stimulate PRMT1 and PRMT5 activity in methylating H4R3 (Beck et al., 2006) *in vitro*. Interestingly, R17 and R19 are located within what is known as the “basic patch” of histone H4 and have been shown to be critical in regulating chromatin dynamics and the binding of multiple chromatin-modifying enzymes (Liu et al., 2017; Meriesh et al., 2020; Zhang X. et al., 2022). It is reasonable to speculate that methylation at these two sites would have a significant impact on chromatin remodeling. A more comprehensive list of histone arginine methylation sites is summarized in Table 2.

**TABLE 2:** Arginine methylation of Histones.

Histone	Site	Types	PRMTs	Function	Refs
H2A	R3me			Fertilization–Transcription Repression	Sarmento et al. (2004)
	R11me1/2	ADMA	PRMT1,6	Chromatin Compaction–Transcriptional Repression	Waldmann et al. (2011), Macadangdang et al. (2014)
	R20me1			unknown	Guo et al. (2014)
	R29me1/2	MMA, ADMA	PRMT6	Transcriptional Repression	Waldmann et al. (2011)
	R42me1			unknown	He et al. (2021)
	R71me1			unknown	Tweedie-Cullen et al. (2012), Boyer et al. (2020)
	R88me1			Sperm differentiation	Shrestha et al. (2022), Blanco et al. (2023)
H2B	R79me1			unknown	He et al. (2021)
	R86me1			unknown	Sylvestersen et al. (2014)
	R92me1			unknown	Jufvas et al. (2011)
	R99me1			unknown	Zhang et al. (2003)
H3	R2me	MMA	PRMT7	Transcriptional activation	Kirmizis et al. (2009)
	R2me2s	SDMA	PRMT5	Transcriptional activation–correlate with H3K4me3	Yuan et al. (2012), Morita et al. (2021)
	R2me2a	ADMA	PRMT6	Transcription Repression–Chromatin condensation	Kim et al. (2020b)
	R8me1	MMA	PRMT7	Transcriptional repression	Chen et al. (2015)
	R8me2a	ADMA	PRMT2	Transcriptional activation	Blythe et al. (2010)
	R8me2s	SDMA	PRMT5	Transcriptional repression	Pal et al. (2004)
	R17me2a	ADMA	CARM1	Transcriptional activation	Daujat et al. (2002), An et al. (2004), Yue et al. (2007)
	R26me2a	ADMA	CARM1	Transcriptional activation	Guccione et al. (2007)
	R42me2a	ADMA	CARM1, PRMT6	Transcriptional activation–p53 dependent	Casadio et al. (2013)
	R52me1			unknown	Hyland et al. (2005)
	R53me1			unknown	Hyland et al. (2005)
	R63me1			unknown	He et al. (2021)
	R83me1			Sperm cell specific	Brunner et al. (2014)
	R83me2			unknown	Bremang et al. (2013)
	R128me1			unknown	Tan et al. (2011), Sundar et al. (2014)
H4	R3me	MMA	PRMT7	Transcriptional activation–Foxm1	He et al. (2021)
	R3me2s	SDMA	PRMT5	Transcriptional repression	Zhao et al. (2009)
	R3me2a	ADMA	PRMT1	Transcriptional activation	Wang et al. (2001)
	R17me1	MMA	PRMT7	Transcriptional repression–in cooperation with PRMT5	Jain et al. (2017)
	R17me2			unknown	Tweedie-Cullen et al. (2012)
	R19me1	MMA	PRMT7	Transcriptional repression–in cooperation with PRMT5	Jain et al. (2017)
	R19me2			unknown	Tweedie-Cullen et al. (2012)
	R35me1			unknown	Sundar et al. (2014)
	R55me1			unknown	Beck et al. (2006), Sundar et al. (2014)
	R67me1			unknown	He et al. (2021)
	R92me1			unknown	Hyland et al. (2005)

### Arginine methylation of epigenetic modifiers

In addition to arginine methylation, histones are also subjected to other types of PTMs, such as acetylation and lysine methylation (Huang et al., 2014; Millan-Zambrano et al., 2022). Many of the epigenetic modifiers that deposit these modifications are regulated by arginine methylation, which in turn impacts histone codes. For example, the histone methyltransferase EZH2 is methylated by PRMT1 at R342 (Li et al., 2020a; Li et al., 2020b; Li et al., 2021). R342me2a inhibits the CDK1-mediated EZH2 phosphorylation at T345 and T487, which otherwise would promote EZH2 ubiquitination and degradation (Li et al., 2020b). On the other hand, the same R342me2a was shown to prevent AMPK-mediated EZH2 phosphorylation at T311 to enhance EZH2 interaction with SUZ12 and to promote H3K27me3 deposition (Li et al., 2021). Interestingly, PRMT5 has also been shown to either associated with EZH2 or impact H3K27me3, although whether there is a direct methylation of EZH2 by PRMT5 is not clear (Liu F. et al., 2020; Yang et al., 2021). Ash2L is a shared component of mammalian histone H3K4 methyltransferase complexes, which include the mixed lineage leukemia (MLL) family members 1–4, Setd1A, and Setd1B, and is essential to maintain global H3K4me3 levels (Milne et al., 2002). Both PRMT1 and PRMT5 can methylate Ash2L at R296. Although R296 methylation is not required for Ash2L nuclear localization or global H3K4me3 levels, it remains unchecked if this modification affects locus-specific H3K4me3 deposition. Additionally, lysine demethylase LSD1 is also a substrate of arginine methylation (Liu et al., 2020b). CARM1 catalyzes LSD1 methylation at R838, which promotes the binding of the deubiquitinase USP7, resulting in the deubiquitination and stabilization of LSD1. Consequently, two of LSD1 substrates, H3K4me2 and H3K9me2, were affected (Liu et al., 2020b). Interestingly, PRMT5 was shown to cooperate with LSD1 in Slug-mediated transcription activation through histone arginine methylation (Zhang J. et al., 2022), whether PRMT5 can methylate LSD1 has not been tested. Altogether, these studies highlighted a broad impact of PRMTs in histone modifications and epigenetic regulation beyond its direct activity on histone arginine methylation.

### Arginine methylation of chromatin remodeling factors

SWI/SNF is a multisubunit chromatin-remodeling complex that utilizes ATP to alter high-order chromatin structures for transcription regulation (Mathur and Roberts, 2018; Centore et al., 2020). One of the core subunits BAF155 is methylated by PRMT4/CARM1 (Wang et al., 2014; Kim et al., 2021). Significantly, CARM1 methylates BAF155 at a single site R1064, and majority of endogenous BAF155 carries R1064me2a, indicating a critical function of this methylation site in BAF155 chromatin remodeling activity (Wang et al., 2014; Kim et al., 2021). The nucleosome remodeling and deacetylase (NuRD; also known as Mi-2) complex is another group of multisubunit ATP-dependent chromatin remodeling complexes with histone deacetylase activity (Lai and Wade, 2011). The non-enzymatic subunits, including methyl-CpG-binding domain 2 (MBD2) and MDB3, are important for targeting NuRD complex to regions with methylated DNA (Lai and Wade, 2011). MBD2/NuRD and MBD3/NuRD were shown to have different biochemical and functional properties–PRMT5 and its cofactor MEP50 were identified as specific MBD2/NuRD components (Le Guezennec et al., 2006). PRMT5 methylates the RG rich region of MBD2, which reduces its interaction with methylated DNA and subsequently the recruitment of histone deacetylases (Tan and Nakielny, 2006). Thus, MBD2 methylation by PRMT5 inhibits the repressive function of the NuRD complex. Additionally, the Bromodomain-containing protein 4 (BRD4), a chromatin-binding protein that functions as a scaffold for transcription factors at promoters and super-enhancers, is methylated by PRMT2 and PRMT4/CARM1 at R179, 181, and 183 upon DNA damage (Liu L. et al., 2022). Methylation selectively controls a transcriptional program by promoting BRD4 recruitment to acetylated histones/chromatin. BRD4 has been shown to harbor histone acetyltransferase activity and can evicts nucleosomes from chromatin (Devaiah et al., 2016). Thus, arginine methylation might impact its function in chromatin remodeling.

### Arginine methylation in the regulation of epitranscriptomics

Another research area of gene regulation that arises in recent years is the chemical modification of RNA molecules (Song and Yi, 2017). In the last two decades, with the advance of technologies in deep sequencing and mass spectrometry, hundreds of modifications have been identified across all RNA species and domains of life (Frye et al., 2016). These RNA post-transcriptional modifications are collectively called epitranscriptomics (Meyer and Jaffrey, 2014; Wiener and Schwartz, 2021). Among them, N6-Methyladenosine (m^6^A) is the most abundant internal modification of mRNA and involves in every step of mRNA life cycle, including splicing, translation, and stability (He and He, 2021). Additionally, m^6^A modification of chromosome-associated regulatory RNA (carRNA), such as promoter-associated RNAs, enhancer RNAs, and repeat RNAs, play critical roles in controlling their expression levels, and consequently the chromatin state and transcription (Liu et al., 2020c; Huang et al., 2020). Majority of mRNA m^6^A is catalyzed by a multicomponent methyltransferase complex containing the methyltransferase-like 3 (METTL3)/methyltransferase-like 14 (METTL14) heterodimer, and other regulatory factors (Wang P. et al., 2016). Three studies have independently reported arginine methylation of METTL14 by PRMT1 (Liu et al., 2021; Wang et al., 2021; Wang et al., 2023), all of which revealed a positive regulation of m^6^A methyltransferase activity by arginine methylation. Although these studies focused on m^6^A on mRNA, reduced methyltransferase activity could also cause decrease of m^6^A levels in carRNA, which might provide a novel functional role of arginine methylation in chromatin biology.

## Regulation of arginine methylation

Recombinant PRMTs purified from bacteria are biochemically active and share many common substrates *in vitro*. Thus, to ensure their biological function *in vivo*, proper regulatory mechanisms are imperative for these otherwise promiscuous enzymes. PRMTs undergo regulation at various levels, including isoform-specific gene expression, subcellular compartmentalization, substrate recognition, and modulation of catalytic activity, among others. These multifaceted regulatory processes collectively contribute to the precise control and effective functioning of PRMTs within living organisms.

### Regulation of PRMT expression

At the RNA level, the expression of PRMTs can be regulated through alternative splicing. Although the functional significance of many isoforms has yet to be determined due to their low abundance, a few do exhibit distinct biological characteristics. For example, next-generation sequencing (NGS) identified more than 30 protein coding isoforms of PRMT1 (Adamopoulos et al., 2019), some of which are associated with specific substrates and unique nucleocytoplasmic patterns (Goulet et al., 2007). The majority of studies have focused on the most abundant isoform, PRMT1v1, which has a broad substrate spectrum in both nuclear and cytoplasm. The v2 isoform differs from v1 by harboring an additional 18-amino acid sequence of a nuclear export sequence and thus is exclusively localized in the cytoplasm. PRMT1v2 exhibits oncogenic function by promoting the survival and invasiveness of breast cancer cells (Baldwin et al., 2012), suggesting that cytoplasmic PRMT1 is sufficient for promoting tumorigenesis. The splicing of PRMT5 is uniquely regulated by a novel class of splicing regulatory elements located between the polypyrimidine tract (Py) and 3′AG (REPA) at intron ends (Sohail et al., 2014). This cis-regulatory element functions as a silencer by binding to hnRNPH and competing off the interaction of U2AF65 with the Py, thus leading to exon skipping. The product of this alternative splicing, PRMT5s, misses 44 amino acids located at the N-terminus of the full-length protein without affecting overall PRMT5 enzyme activity (Sohail and Xie, 2015). However, the PRMT5s exhibits drastic differences in subcellular localization, indicating that it might methylate different set of substrates (Sohail et al., 2015; Sohail and Xie, 2015; Wen et al., 2022). Additionally, knockdown the key splicing factor SF3B1 leads to the exon 5 skipping of PRMT9 (Choi et al., 2020), which could lead to translation frame shift and reduce PRMT9 expression through nonsense-mediated RNA decay.

### Subcellular compartmentalization

The best example for this type of regulation is demonstrated on PRMT8, which harbors an N-terminal myristoylation site that directs PRMT8 to the plasma membrane. The expression of PRMT8 is mainly found in the brain (Lee J. et al., 2005; Kousaka et al., 2009), suggesting its function in methylating membrane associated proteins and involving in signaling transduction (Kim et al., 2008; Pahlich et al., 2008). Interestingly, the enzymatic activity and the interaction with its methylation substrates are necessary for PRMT1 and PRMT3’s subcellular localization. Catalytic inactive PRMT1 accumulates in the nucleus (Herrmann and Fackelmayer, 2009), and treatment of cells with a global methyltrasferase inhibitor, AdOx, causes PRMT3 (a cytoplasmic protein) to be nuclear (Xie and Denman, 2011). Consistent with these earlier reports, a recent study revealed that phosphorylation of PRMT1 at S307 by the cyclin-dependent kinase 5 (CDK5) promotes its translocation from nucleus to cytoplasm, which is associated with an enhanced methyltransferase activity (Yin et al., 2023). Furthermore, during the specification of primordial germ cells (PGCs) in mice, PRMT5 is in complex with the SET–PR domain protein Blimp1, also known as PRDM1, in the nucleus at the embryonic day 8.5 (E8.5) and subsequently translocated to the cytoplasm at E11.5. Although the molecular mechanism underlying this nuclear-cytoplasm translocation is unknown, this process could be important for the extensive epigenetic programming during germ cell development (Ancelin et al., 2006). Notably, specific subcellular compartmentalization of PRMTs has been associated with the specific subtypes of cancer and the progression of the malignancy (Davis et al., 2013; Shilo et al., 2013), highlighting the prognostic value for understanding this type of regulation in cancer treatment.

### PTMs of PRMTs

PTMs are extensively identified on PRMTs and have emerged as another key layer of PRMTs’ regulation. The impacts of these modifications, including phosphorylation (auto)methylation, OGT-glycosylation, and ubiquitination, on various aspects of PRMTs’ function were recently reviewed (Hartley and Lu, 2020). Here, we added a few examples that were not previously covered. Whereas polyubiquitination of PRMT5 by the E3 ligase CHIP was found to negatively regulate PRMT5 protein expression through K48-linked ubiquitin-dependent proteasomal degradation (Zhang et al., 2016), PRMT5 is also a substrate of TRAF6, which deposits K63-linked ubiquitin chain (Liu et al., 2023). Multiple K63 ubiquitination sites were identified at the N-terminus of PRMT5, which enhances its interaction with co-factor MEP50 and its enzymatic activity (Liu et al., 2023). Note that the K48 ubiquitination sites are located in the central region of PRMT5, away from the K63 ubiquitination sites (Zhang et al., 2016).

### Substrate recognition of PRMTs

PRMTs do not contain any specific domains for DNA or histone interaction. Their functions in transcription and epigenetic regulation are largely achieved by the recruitment through protein–protein interactions with transcription factors and co-regulators. These interactions significantly contribute to their substrate specificity. For example, PRMT5 interacts with the hSWI/SNF chromatin remodeler complex, which in turn enhances its methyltransferase activity towards histone substrates (An et al., 2004). The histone-binding protein COPR5 specifically interacts with the N-terminus of histone H4 and guides PRMT5 to methylate H4R3 rather than H3R8 (Lacroix et al., 2008). Recombinant PRMT4/CARM1 preferentially methylates free histone 3, whereas upon association with the nucleosomal methylation activator complex (NUMAC), PRMT4/CARM1 obtains the activity to methylate nucleosome histone 3 (Xu et al., 2004). Another important layer of regulation on PRMT’s substrate recognition resides the sequence context of the substrates, specifically methylation site could be influenced by other PTMs in close proximity. Such regulations are prevalent on histone substrates because of their extensive PTMs. For example, PRMT4/CARM1 prefers Histone H3 lysine 18 acetylation (H3K18ac) modified histone for depositing methylation mark at arginine 17 residue (H3R17me2a) (Daujat et al., 2002; Guccione et al., 2007; Yue et al., 2007). H3K9ac impedes PRMT5 for the deposition of H3R8me2s mark (An et al., 2004). Histone H4 lysine 5 acetylation (H4K5ac) primes the H4R3 site for methylation by PRMT5, but not PRMT1, and consequently switching an active mark to a repressive mark (H4R3me2s) (Feng et al., 2011). At the CTD of RNAPII, phosphorylation of either Serine 2 or Serine 5 within the heptameric repeats could block the methylation of R1810 by CARM1 (Mizutani et al., 2015). Furthermore, in glioblastoma, 5-hydroxymethylcytosine (5 hmC) mark on DNA can recruit PRMT1 to chromatin by interacting with an accessory protein of PRMT1, chromatin target of PRMT1 (CHTOP), linking the modification of DNA with histone methylation (Takai et al., 2014). Additional examples of this regulation can be found in the “Arginine methylation crosstalk with other PTMs” section.

### Regulation of PRMT activity by cellular metabolites

S-adenosylmethionine (SAM) is the universal donor for all methylation reactions, including DNA, RNA, and protein methylation (Mentch et al., 2015). It is catalyzed from amino acid methionine and ATP by methionine adenosyl transferase enzymes (MATs), which are encoded by the MAT1A and MAT2A genes (Cantoni, 1953). In the methionine salvage pathway, the methylthioadenosine phosphorylase (MTAP) cleaves the byproduct of polyamine biosynthesis, methylthioadenosine (MTA), into adenine and MTR-1-P (5-methythioribose-1-phosphate), which eventually leads to the regeneration of methionine (Zappia et al., 1988). Additionally, methylation reaction generates S-adenosylhomocysteine (SAH) as a byproduct that needs to be hydrolyzed to homocysteine (Hcy) and adenine by S-adenosylhomocysteine hydrolase (AHCY) (Obeid and Herrmann, 2009). Cellular accumulation of SAH and MTA, in principle, could suppress all methylation reactions. For example, deletion of MTAP, which occurs in approximately 15% of human cancers causes the accumulation of MTA that imposes the strongest inhibitory effect on PRMT5. Thus, MTAP deleted tumors are highly sensitive to further inhibition of PRMT5 activity (Kryukov et al., 2016; Mavrakis et al., 2016). On top of MTAP deletion, inhibition of MAT2A, which reduces available SAM, further strengthens PRMT5 dependency (Marjon et al., 2016). Thus, PRMT5 inhibitor exhibited highest efficacy in tumor suppression under this context (Fedoriw et al., 2019; Marjon et al., 2021). Mechanistically, PRMT5 seems to be the most sensitive one among many protein methyltransferases upon MTA inhibition (Kryukov et al., 2016). On the other hand, fluctuations in SAM level could also differentially impact PRMTs activity due to their different apparent *K*m for SAM. Among all PRMTs, PRMT9 has the highest SAM *K*m (40.5 ± 1 μM), which is 10-20 fold higher than other PRMTs (Li A. S. M. et al., 2020), suggesting that PRMT9 might be a frontline sensor for SAM alternation in response to methionine metabolism.

## Future perspectives

### Arginine demethylases

Dynamic arginine methylation and demethylation play crucial roles in the regulation of protein function. In comparison to the well-characterized writers and readers of arginine methylation, the biochemical properties and biological functions of erasers or demethylases that reverse the methylation reaction remain elusive. However, it becomes clear that lysine demethylases are capable of catalyzing arginine demethylation *in vitro* (Walport et al., 2016). These enzymes belong to 2-Oxoglutarate-dependent dioxygenases, which require oxygen, reduced iron and 2-oxoglutarate (also known as *α*-ketoglutarate) as cofactors to function (Herr and Hausinger, 2018; Islam et al., 2018; Losman et al., 2020). Thus, when these metabolites become limited, their enzymatic activities are often inhibited. Although this connection has been well documented with histone lysine methylation marks, such as H3K4me3 and H3K27me3 (Mentch et al., 2015; Pan et al., 2016; Batie et al., 2019; Chakraborty et al., 2019), whether and how metabolic alterations impact histone arginine methylation is unknown. Furthermore, if demethylation of lysine and arginine share the same family of demethylases, what are the factors that determine their substrate selectivity and specificity, if any? As the levels of histone lysine methylation are in general higher than that of arginine methylation (Huang et al., 2015), the availability and concentrations of specific co-factors might differentially influence the enzymatic activity and substrate specificity. The surrounding amino acid sequence and structural context of the target residue could also differentially influence the substrate recognition by demethylases. Understanding the precise mechanisms of substrate selectivity and specificity of lysine and arginine demethylases will provide more comprehensive understanding of epigenetic regulation through these two types of histone marks.

### Tudor domain inhibitors

Targeting protein–protein interactions (PPIs) for drug discovery are considered challenging because PPIs are often characterized by large and flat protein surfaces, making it difficult to design small molecules that can effectively bind and disrupt the interaction (Scott et al., 2016). Recently, the development of chemical inhibitors that target bromodomain and extra-terminal domain (BET) containing proteins has gained significant attention in the field of cancer research. These inhibitors, represented by JQ1, competes with acetylated lysine residues for binding to the bromodomain pockets of BET proteins, thus inhibiting their transcriptional activity (Filippakopoulos et al., 2010; Dawson et al., 2011; Delmore et al., 2011; Mertz et al., 2011). A few inhibitors have been developed to target methyl lysine binding Tudor domains, such as those of UHRF1 and SETDB1 (Senisterra et al., 2018; Chang et al., 2021; Guo et al., 2021), whereas the development of methylarginine Tudor domain inhibitors is lagging. The SMN Tudor-selective antagonist was recently discovered by serendipity while searching for inhibitors against the histone H3K9me3 binding tandem Tudor domain (TTD) of UHRF1 (Liu Y. L. et al., 2022). This inhibitor binds to Tudor domain of SMN, SMNDC1, and TDRD3 with slightly higher affinity than to UHRF1 (Liu Y. L. et al., 2022). In cells, it inhibits SMN interaction with RNAPII and causes transcription termination, mirroring the effect of SMN knockdown (Liu Y. L. et al., 2022). However, its selectivity for SMN is only ∼4-fold over TDRD3. Thus, the observed *in vivo* effect could partially contribute from TDRD3 inhibition. Using nuclear magnetic resonance (NMR) based fragment screening, Liu et al. identified several small molecule inhibitors of TDRD3 Tudor domain with a *K*d of ∼50 uM. These compounds show reduced binding to SMN Tudor domain and no detectable binding with the methyl lysine binding Tudor domain of 53BP1 (Liu et al., 2018). Although the *in vivo* efficacy of these inhibitors has yet been determined, this study provided useful information for future structure-guided discovery of Tudor domain inhibitors.
